# Progressive suppression of DNA repair genes with persistent p53 target activation in doxorubicin-treated cardiomyocytes

**DOI:** 10.1093/g3journal/jkag144

**Published:** 2026-06-22

**Authors:** Emma M Pfortmiller, José A Gutiérrez, Alyssa R Bogar, Michelle C Ward

**Affiliations:** Pharmacology and Toxicology Graduate Program, The University of Texas Medical Branch at Galveston, Galveston, TX 77555, United States; Department of Biochemistry and Molecular Biology, The University of Texas Medical Branch at Galveston, Galveston, TX 77555, United States; Department of Biochemistry and Molecular Biology, The University of Texas Medical Branch at Galveston, Galveston, TX 77555, United States; Department of Biochemistry and Molecular Biology, The University of Texas Medical Branch at Galveston, Galveston, TX 77555, United States

**Keywords:** DNA damage, Cardiomyocytes, Doxorubicin, Global gene expression

## Abstract

Doxorubicin (DOX) is an effective anticancer drug; however, it can cause cardiotoxicity by inducing DNA double-strand breaks in cardiomyocytes. Cardiotoxicity can manifest immediately or years following treatment. Most human in vitro models of DOX-induced cardiotoxicity (DIC) focus on the acute effects of DOX treatment. To understand the long-term effects, we profiled the global gene expression response to DOX exposure over time. We treated iPSC-derived cardiomyocytes from six individuals with DOX for 24 h and assayed responses after 0, 24, and 144 h of recovery. DNA damage, determined by γH2AX expression, is induced following DOX treatment and is resolved by the final recovery timepoint. We identified both acute and chronic gene expression response signatures. The chronic signature, representing 501 genes, is enriched for p53 target genes, DNA damage response (DDR) genes, and senescence-associated genes compared to acute response genes. p53 target genes are persistently activated, and DDR genes are progressively downregulated over time. Our results suggest an altered cell state following repair of double-strand breaks that is distinct from preexposed cells. Doxorubicin response genes with persistent changes in expression can be applied to the design of toxicity biomarkers or therapeutic targets.

## Introduction

Doxorubicin-induced cardiotoxicity (DIC) is a well-established clinical phenomenon that affects a subset of cancer patients treated with the drug (Camilli et al. 2024). Cardiotoxicity can lead to cardiac dysfunction and ultimately heart failure (Cardinale et al. 2020). Doxorubicin-induced cardiotoxicity can occur soon after or years following treatment. Typically, cardiotoxicity is diagnosed once damage has occurred. For example, elevated levels of cardiac troponin I in the blood indicate that the heart is under stress. Cardioprotective agents can be administered to prevent cardiotoxicity; however, there is currently only one FDA-approved drug, dexrazoxane, that is in limited use given its reduced anticancer efficacy. There is therefore a need to identify both early biomarkers of toxicity and potential therapeutic targets.

Doxorubicin (DOX) is a topoisomerase II inhibitor (TOP2i) that induces DNA double-strand breaks. The molecular basis of DIC is challenging to study in humans in vivo given the inaccessibility of the heart muscle. Induced pluripotent stem cell-derived cardiomyocytes (iPSC-CMs) have been shown to effectively model the disease (Burridge et al. 2016). Treating iPSC-CMs with sub-micromolar concentrations of DOX induces large scale changes to the transcriptome (Knowles et al. 2018; Matthews et al. 2024) and the proteome (Johnson et al. 2025). Induced pluripotent stem cell-derived cardiomyocytes have also been used to identify mediators of DOX toxicity using functional genomic CRISPR screens, which have highlighted the involvement of transporters such as SLC01A2 and metabolic enzymes such as carbonic anhydrase (Sapp et al. 2021; Liu et al. 2024). CRISPR knockout of genes in genetic loci associated with DIC have also highlighted genes most likely to be associated with the disease (Fonoudi et al. 2024). All the studies described above measure the effects of DOX within 72 h of treatment.

Clinically, DOX is typically administered through weeklong cycles which include recovery periods between treatments. Human autopsies of individuals treated with DOX antemortem have detectable levels of DOX in their heart tissue suggesting that long-term exposure of cardiomyocytes may have long-term effects (Stewart et al. 1993). Chronic DOX treatments have been administered in mouse and rat in vivo models of cardiotoxicity (Zhu et al. 2008; Podyacheva et al. 2021); however, these studies are unable to detect human-specific responses. A repeated exposure framework has been implemented in studies using iPSC-CMs from a single individual which have identified effects on miRNA expression, metabolites, and cardiomyocyte contraction indicating likely dynamics in treatment responses (Chaudhari et al. 2016a, 2016b; Chaudhari et al. 2017).

To capture a chronic rather than acute DOX exposure state in human cardiomyocytes that can contribute to the development of biomarkers, we designed a study to identify gene expression changes that persist in iPSC-CMs following DOX exposure. We treated iPSC-CMs from six individuals with a sublethal DOX concentration for 24 h and measured the effects 0, 24, and 144 h following the removal of the drug. This allowed us to identify both acute and chronic gene expression patterns and characterize their properties.

## Methods

### Induced pluripotent stem cell lines

Induced pluripotent stem cells were sourced from the iPSC Collection for Omic Research (iPSCORE) Resource, generated by Dr. Kelly A. Frazer at the University of California San Diego as part of the National Heart, Lung, and Blood Institute Next-Gen Consortium (Panopoulos et al. 2017). Induced pluripotent stem cell lines, reprogrammed from skin fibroblasts, may be obtained through contacting Dr. Kelly A. Frazer at the University of California San Diego or through the biorepository at the WiCell Research Institute (Madison, WI, USA).

We utilized six human iPSC lines derived from three males and three females between the ages of 18 and 32 years. These individuals are unrelated and have no prior history of disease. Individual details: Individual 1: UCSD143i-87-1, female, age 21, Asian (Chinese); Individual 2: UCSD132i-78-1, female, age 21, Asian (Chinese); Individual 3: UCSD129i-75-1, female, age 30, Asian (Irani); Individual 4: UCSD138i-84-1, male, age 21, Asian (Chinese); Individual 5: UCSD178i-17-3, male, age 18, Asian (Japanese); Individual 6: UCSD154i-90-1, male, age 23, Asian (Chinese).

### iPSC maintenance

Induced pluripotent stem cells were cultured in feeder-free conditions at 37 °C with 5% CO_2_ and atmospheric oxygen. Induced pluripotent stem cells were maintained with mTESR1 (85850, Stem Cell Technology, Vancouver, BC, Canada) media with 1% penicillin/streptomycin (30-002-Cl, Corning, Bedford, MA, USA) on Matrigel matrix (354277, Corning, Bedford, MA, USA) at a 1:100 dilution. Induced pluripotent stem cells were passaged every 3 to 5 days as they reached a confluency of 60% to 80%.

### Cardiomyocyte differentiation

Induced pluripotent stem cells were differentiated into cardiomyocytes as previously described (Matthews et al. 2024). Induced pluripotent stem cells were seeded onto a Matrigel-coated 100 mm cell culture dish and maintained until reaching 85% to 90% confluency. The initial step to begin differentiation (Day 0) includes adding the GSK3 inhibitor CHIR99021 trihydrochloride (4953, Tocris Bioscience, Bristol, UK) in cardiomyocyte differentiation media (CDM) containing 500 mL RPMI 1640 (15-040-CM, Corning, Bedford, MA, USA), 10 mL B-27 minus insulin (A1895601, ThermoFisher Scientific, Waltham, MA, USA), 5 mL of GlutaMAX (35050-061, ThermoFisher Scientific), and 5 mL of penicillin/streptomycin (100X) (30-002-CI, Corning) to yield a final concentration of 12 μM. Media was replaced with CDM after 24 h (Day 1). After 48 h (Day 3), 2 μM of the Wnt signaling inhibitor Wnt-C59 (5148, Tocris Bioscience) was added to cells in CDM. Cardiomyocyte differentiation media was replaced on Days 5, 7, 10, and 12. Cells start spontaneously contracting between Day 7 and 10. Induced pluripotent stem cell-derived cardiomyocytes were selected using lactate-containing, glucose-free purification media (500 mL RPMI without glucose [11879, ThermoFisher Scientific], 106.5 mg L-ascorbic acid 2-phosphate sesquimagnesium salt [sc228390, Santa Cruz Biotechnology, Santa Cruz, CA, USA], 3.33 mL 75 mg/mL human recombinant albumin [A0237, Sigma-Aldrich, St Louis, MO, USA], 2.5 mL 1 M lactate in 1 M HEPES [L(+)Lactic acid sodium {L7022, Sigma-Aldrich}], and 5 mL penicillin/streptomycin [100X] [30-002-CI, Corning]). Purification media was replaced on Days 14, 16, and 18. On Day 20 purified iPSC-CMs were dissociated from the culture plate with 0.05% trypsin/0.53 mM EDTA (MT25052CI, Corning) and counted using a Countess 2 machine. Induced pluripotent stem cell-derived cardiomyocytes were plated in a galactose-containing, glucose-free cardiomyocyte maintenance media (CMM) (500 mL DMEM without glucose [A14430-01, ThermoFisher Scientific], 50 mL FBS [MT35015CV, Corning], 990 mg galactose [G5388, Sigma-Aldrich], 5 mL 100 mM sodium pyruvate [11360-070, ThermoFisher Scientific], 2.5 mL 1 M HEPES [H3375, Sigma Aldrich], 5 mL 100× GlutaMAX [35050-061, ThermoFisher Scientific], and 5 mL penicillin/streptomycin [100X] [30-002-CI, Corning]) to metabolically mature iPSC-CMs. Induced pluripotent stem cell-derived cardiomyocytes were maintained in culture for an additional 5 to 10 days. Cardiomyocyte maintenance media was replaced on Days 23, 25, 27, and 29.

### Cardiac troponin T flow cytometry for iPSC-CM purity determination

Following iPSC-CM differentiation, culture purity was assessed using flow cytometry for cardiac troponin T (TNNT2) expression. Day 25 ± 1 iPSC-CMs were released from the cell culture plate using 0.05% trypsin/0.53 mM EDTA (MT25052CI, Corning) and quenched with CMM. Cell suspensions were strained to generate single cells. A total of 3 million cells were divided into one sample well (TNNT2 and live/dead stain) and three control wells (TNNT2-only, live/dead stain-only and unlabeled) in a deep-well U-bottom plate (13-882-234, BrandTech Scientific, Essex, CT, USA). Cells were stained with a viability marker, Zombie Violet Fixable Dye, diluted in PBS (423113, BioLegend, San Diego, CA, USA) for 30 min at 4 °C in the dark. Cells were washed with PBS and then autoMACS running buffer (130-091-221, Miltenyi Biotec, San Diego, CA, USA). Cells were fixed and permeabilized using the FOXP3/Transcription Factor Staining Buffer Set (00-5523, ThermoFisher Scientific) for 30 min at 4 °C in the dark. Cells were washed and stored in autoMACS running buffer O/N. Cells were incubated with 5 μL Cardiac Troponin T Mouse, PE, Clone: 13-11, BD Mouse Monoclonal Antibody (564747, BD Biosciences, San Jose, CA, USA) diluted in permeabilization buffer from the FOXP3/Transcription Factor Staining Buffer Set (00-5523, ThermoFisher Scientific) for 40 to 50 min in the dark at 4 °C. Cells were washed twice with permeabilization buffer and resuspended in autoMACS buffer. A BD LSR Fortessa Cell Analyzer was used to analyze 10,000 cells per sample.

The proportion of live cells expressing TNNT2 was calculated as follows: (i) Cellular debris was excluded by adjusting forward and side scatter. (2) Single-celled populations were gated by forward scatter height and area. (iii) The Zombie Violet-positive population was excluded as dead cells. (iv) TNNT2 positivity in the live population was determined based on the unlabeled cell population.

### Drug stock preparation

Doxorubicin hydrochloride (D1515; Sigma-Aldrich) was dissolved in DMSO to create a 10-mM stock solution. Aliquots of 100 μL were prepared in separate tubes and stored at −80 °C. A working stock of the desired concentration was prepared in CMM immediately preceding treatment. All DMSO vehicle (VEH) control treatments were prepared using identical volumes to the DOX working stock.

### PrestoBlue cell viability assay

Three individuals (Individuals 2, 3, and 5) were utilized to perform cell viability assays on Day 32 to 35 iPSC-CMs. Six 96-well plates were seeded with 50,000 iPSC-CMs per well excluding the outer wells. Prior to treatment, a baseline cell viability measurement (time 0) was taken using the PrestoBlue cell viability assay (A13262, Invitrogen, Waltham, MA, USA) according to manufacturer instructions with a 1-h incubation period. Induced pluripotent stem cell-derived cardiomyocytes were treated with a range of eight DOX concentrations (0.01 to 1000 μM) and volume-matched VEH in quadruplicate for 24 h. Controls included eight CMM-only iPSC-CM wells per timepoint and six cell-free wells per plate. Treatments were randomized with the Well Plate Maker (wpm) package in R (Borges et al. 2021). Viability was determined with the PrestoBlue assay 24 h following treatment (time 24). Fluorescence was determined using a Biotek Synergy H1 plate reader with an excitation/emission of 460/490 nm.

To determine cell viability at each time point, background fluorescence was first averaged from the six cell-free wells and subtracted from all samples. This yielded relative fluorescence units (RFLU) for each sample. Relative fluorescence unit values from samples on each plate were divided by the CMM-only iPSC-CM wells and normalized by the average DOX concentration-matched VEH wells. Time 24 data was divided by time 0 data to yield a relative percent cell viability. A dose–response curve for each individual was generated using the drc package in R which calculated LD50 values for each individual at each timepoint (Ritz et al. 2015).

### γH2AX and Hoechst immunofluorescence staining and quantification

Induced pluripotent stem cell-derived cardiomyocytes for three individuals (Individual 1, 3, and 4) were plated at 150,00 cells per well in 24-well plates. To enable comparison of γH2AX staining across time, we maintained the time of staining constant and varied the starting treatment time. First, Day 27 iPSC-CMs were treated with 0.5 μM DOX and VEH in duplicate for 24 h and then incubated in CMM only for the next 144 h (tx + 144). All remaining wells received CMM only. On Day 29, CMM was added to all wells. On Day 32, four untreated wells were treated with DOX or VEH in duplicate for 24 h and then recovered for 24 h in CMM (tx + 24). All remaining wells received CMM. On Day 33, four remaining wells were treated with DOX and VEH in duplicate for 24 h (tx + 0). On Day 34, immunofluorescence staining was performed on all treatment timepoints tx + 0, tx + 24, tx + 144, and secondary antibody-only control wells. Induced pluripotent stem cell-derived cardiomyocytes were fixed in 4% paraformaldehyde for 15 min at room temperature. Next, cells were permeabilized for 10 min in 0.25% DPBS-T (0.25% Triton X-100 in DPBS) and washed with cold DPBS. Cells were blocked in 5% BSA in DPBS-T for 30 min at room temperature and incubated overnight at 4 °C with anti-phospho-Histone H2A.X (Ser139) rabbit monoclonal antibody (1:500; NC1602516; Fisher Scientific) prepared in 1% BSA in DPBS-T. Cells were washed with cold DPBS and incubated with a Donkey anti-Rabbit Alexa Fluor 594-conjugated secondary antibody (1:1000; A21207, Invitrogen) for 1 h. Nuclei were counterstained with Hoechst 33342 (PI62249; Thermo Scientific) for 10 min in the dark. Fluorescence imaging was performed on an EVOS microscope. Exposure settings were standardized across all conditions.

#### γH2AX quantification

Quantification of γH2AX positive nuclei was performed using the Cell Counter plug-in in Fiji/ImageJ version 1.54i (Schindelin et al. 2012) and a custom ImageJ macro for agnostic, automated quantification of γH2AX nuclear coverage. At least 100 nuclei were counted across three fields of view across duplicate wells per condition. γH2AX presence was scored across nuclei. γH2AX Texas Red pixel intensity was measured within nuclei and divided by nucleus area to yield normalized γH2AX intensity per nucleus. Within each image, the mean of γH2AX intensity across all nuclei was calculated. Three images with consistent nuclei counts were selected for each condition. Intensities were normalized across images by dividing by the highest intensity measurement in each image. The mean γH2AX intensity across these three images were calculated to yield individual-level γH2AX mean intensity. A t-test was performed to compare γH2AX between DOX- and VEH-treated iPSC-CMs across three individuals at each timepoint. *P* < 0.05 is considered significant. To further assess differences in γH2AX expression over time, a t-test was performed between DOX-treated iPSC-CMs at each timepoint. *P* < 0.05 is considered significant.

#### Hoechst intensity quantification

Hoechst staining was quantified utilizing a custom ImageJ macro to measure mean per-pixel intensity within each nucleus. The mean Hoechst intensity was calculated by averaging all nuclei within a given image. Three images with consistent nuclei counts were randomly selected for each condition. Next, the mean intensity across these three images per condition was calculated. Finally, the mean of the image-level mean intensity was calculated to yield individual-level mean Hoechst intensity. A t-test was conducted to compare the mean intensity between DOX- and VEH-treated iPSC-CMs per timepoint from three individuals as well as between each timepoint per treatment. *P* < 0.05 is considered significant.

#### Nuclei quantification

Quantification of iPSC-CM nuclei was performed by a custom macro utilizing the Analyze Particles function in ImageJ. The macro employed an Otsu threshold and watershed segmentation to agnostically count Hoechst-stained nuclei within each image. The mean nuclei count was calculated across three randomly selected images per condition to yield individual-level nuclei counts. A t-test was performed to compare the mean number of nuclei between DOX- and VEH-treated iPSC-CMs across three individuals at each timepoint and between timepoints for each treatment. *P* < 0.05 is considered significant.

### Western blot analysis

Protein was extracted from DOX- and VEH-treated iPSC-CM cell pellets from Individual 1 using RIPA buffer containing EDTA-free protease inhibitors (1187350001, Millipore Sigma). Cells were lysed for 1.5 h at 4 °C, centrifuged at 12,000 rpm for 10 min at 4 °C, and passed through a 20 to 22G needle 10 times. Protein content was assessed with a BCA protein assay kit (23227, Pierce, Thermo Scientific) according to manufacturer's instructions. Eighteen to 20 μg of protein was denatured in 2× Laemmli sample buffer (1610737, BioRad, Hercules, CA, USA) containing a final concentration of 2.5% (v/v) 2-mercaptoethanol (21985023, Gibco, ThermoFisher Scientific) at 95 °C for 5 min prior to gel electrophoresis and transfer to nitrocellulose membranes. Blots were blocked in 5% (w/v) fat-free milk-TBST (50488786, Research Products International Corp, Fisher Scientific) for 1 h and then incubated overnight at 4 °C with primary antibodies directed against β-Actin 1:1000 (4970S, Cell Signaling Technology, Danvers, MA, USA), γH2AX 1:1000 (9718S, Cell Signaling Technology), and p53 1:200 (sc-126, Santa Cruz Biotechnology, Dallas, TX, USA) diluted in 5% fat-free milk-TBST. The blots were then incubated with peroxidase-conjugated secondary antibodies for 1 h at room temperature: Goat anti-Rabbit IgG (32460, Invitrogen) 1:500 for β-Actin, 1:2000 for γH2AX, and 1:500 Goat anti-Mouse IgG (32430, Invitrogen) for p53. After incubation, blots were visualized with ECL Western blotting substrate (32209, Pierce, Thermo Scientific) and imaged on a BioRad ChemiDoc.

### iPSC-CM treatment and collection

1.5 million iPSC-CMs were plated per well of a six-well plate. Day 29 to 30 iPSC-CMs from six individuals were treated with 0.5 μM DOX and matched VEH in CMM across three six-well plates. The differentiation and treatment was replicated in one of the individuals (Individual 6). Following 24 h of treatment, one plate of DOX and VEH was harvested (tx + 0). Cardiomyocyte maintenance media was replaced on the remaining plates. Following 24 h of recovery, one plate of DOX and VEH was harvested (tx + 24). Cardiomyocyte maintenance media was replaced 48, 96, and 144 h after treatment (Day 32 +/−1, Day 34 +/−1, and Day 36 +/−1). Following 144 h of recovery, the final DOX- and VEH-treated plate was collected (tx + 144). At each timepoint, iPSC-CM cells were harvested on ice, flash-frozen, and stored at −80 °C.

### RNA extraction

RNA was extracted from 42 flash-frozen iPSC-CM cell pellets in treatment-balanced batches. All six treatment conditions per individual were extracted in one batch. RNA was extracted across seven batches corresponding to the six individuals and one replicated individual. Total RNA was extracted using the RNeasy Mini Kit 250 (74106; QIAGEN, Germantown, MD, USA) following instructions from the manufacturer. An Agilent 2100 Bioanalyzer was used to evaluate RNA integrity and concentration. All samples exceeded an RNA Integrity Number (RIN) of 8.0.

### RNA-seq library preparation

dT-selected stranded RNA-seq libraries were prepared from 250 ng of total RNA from each sample, with the exception of the six samples from Individual 1 (35 to 169 ng). Poly(A) RNA was isolated using the NEBNext Poly(A) mRNA Magnetic Isolation Module (E7490L, New England Biolabs, Ipswich, MA, USA). RNA-seq libraries were prepared in a single batch using the NEBNext Ultra II RNA Library Prep with Sample Purification Beads 96 reactions kit (E7775 K, New England Biolabs) and then indexed using the NEBNext Multiplex Oligos for Illumina (96 Unique Dual Index Primer Pairs, E6440S). Following library preparation, quality was assessed on an Agilent 2100 Bioanalyzer prior to quantification, pooling, and sequencing. All 42 samples were pooled together and sequenced paired-end 75 bp across two lanes of the Element AVITI (Element Biosciences, San Diego, CA, USA), which yielded a median of 33,462,422 paired-end read fragments across samples.

### RNA-seq analysis

Raw sequencing reads were assessed for quality using FastQC v0.12.1 (Andrews 2010). Reads were aligned to the human hg38 reference genome using Subread (Liao et al. 2013), and read numbers across annotated genes quantified using the featureCounts function (Liao et al. 2014). We transformed raw counts to log_2_ counts per million (log_2_ cpm) using the edgeR package in R (Robinson et al. 2010). Lowly expressed genes with a mean log_2_ cpm < 0 across samples were excluded, resulting in a final set of 14,319 expressed genes.

#### Principal component analysis

Principal component analysis (PCA) was performed on log_2_ cpm values to identify the major contributors to variation in these data. The prcomp function in the stats package v4.4.2 (R Core Team 2024) was used to generate PCA data and visualized with the autoplot function of ggplot2 v3.5.2 (Wickham 2016).

#### Correction for unwanted variation

Factors contributing to unwanted variation were identified using the RUVSeq package in R (Risso et al. 2014). The RUV function was used to estimate factors contributing to variation using the replicated Individual 6 data. Data segregate based on DOX treatment time following correction with one factor.

#### Differential expression analysis

We randomly selected one of the replicated Individual 6 treatment sets and Individuals 1-5 for pairwise differential expression analysis using the edgeR-voom-limma pipeline (Law et al. 2018). We used fixed effects for the treatment, a covariate for the RUV factor, and a random effect for individual implemented through the duplicateCorrelation function. Doxorubicin samples at each timepoint were contrasted with timepoint-matched VEH samples. Genes with a Benjamini–Hochberg corrected *P* value < 0.05 are considered DEGs.

#### Gene expression trajectory analysis

The Cormotif package was utilized to jointly model expression patterns across time (Wei et al. 2015). Cormotif is a Bayesian clustering approach that identifies patterns that best fit the data and defines them as correlation motifs. We used log_2_ cpm of RUVs normalized counts as input and contrasted DOX-VEH pairs across timepoints. Bayesian Information Criterion (BIC) and Akaike Information Criterion (AIC) analysis identified two motifs that best fit the data. We assigned genes to motifs based on their likelihood of belonging to a motif (clustlike) and the posterior probability of being differentially expressed (p.post). Genes were assigned to Motif 1 (Acute) with a clustlike probability > 0.8, p.post > 0.05 in tx + 0, and p.post < 0.5 in tx + 24 and tx + 144 timepoints. Genes were assigned to Motif 2 (Chronic) with a clustlike probability > 0.5, p.post > 0.3 in tx + 0, p.post > 0.5 in tx + 24, and p.post > 0.5 in tx + 144. Using these parameters, 56.9% of all genes are assigned to one of the two motifs.

#### Gene ontology and pathway enrichment analysis

We performed gene ontology analysis for acute and chronic response genes against a background set of all expressed genes. The gProfiler2 tool in R was used to perform gene set enrichment analysis for each gene set (Kolberg et al. 2020). Kyoto Encyclopedia of Genes and Genomes (KEGG) pathways with an adjusted *P* < 0.05 are considered significantly enriched.

#### Comparison to published data

##### TOP2i DEG overlap

We obtained a set of DEGs associated with 24 h of DOX and alternative TOP2i treatments (daunorubicin [DNR], epirubicin [EPI], mitoxantrone [MTX]) from Matthews et al. (Matthews et al. 2024). Of these, 7,703 genes are expressed in our data. In addition, we obtained 278 DEGs associated with CX-5641 treatment for 24 h from Paul et al. (Paul et al. 2026). Of these, 273 are expressed in our data. The proportion of tx + 0 DEGs in each set of TOP2i DEGs was determined. Permutation testing (10,000 iterations) was performed to identify whether the overlap of tx + 0 DEGs was greater than expected by chance using a random sample of expressed genes in our dataset. Significance was determined following multiple testing correction using the Benjamini–Hochberg method. An adjusted *P* < 0.05 is considered significant.

##### DNA damage response gene expression analysis

We obtained a set of 66 DNA damage-associated genes from the Molecular Signatures Database (MSigDB v7.5.1) (Liberzon et al. 2015). Sixty-five genes are expressed in our data. The proportion of DDR genes in each response cluster was determined. A Fisher's exact test was used to compare proportions across correlation motif clusters. *P* < 0.05 is considered significant.

##### P53 target gene expression analysis

We obtained a set of 346 p53 target genes (Fischer 2017). Three hundred genes are expressed in our data. The proportion of p53 target genes in each response cluster was determined. A Fisher's exact test was used to compare proportions across response clusters. *P* < 0.05 is considered significant.

##### Senescence-associated gene expression analysis

We obtained a set of 3,864 unique senescence-associated genes from proliferative cell types, including genes identified in human foreskin fibroblasts (Distefano et al. 2023), human lung epithelial cells, and human lung fibroblasts (DePianto et al. 2021). Of these, 2,497 are expressed in our data. The number of genes present in each response cluster was determined. A Fisher's exact test was performed to compare the proportion of senescence-associated genes across response clusters. *P* < 0.05 is considered significant.

##### DIC gene expression analysis

We compiled a set of 201 unique genes associated with DIC through CRISPR/Cas9 screening (Sapp et al. 2021; Liu et al. 2024) and in vitro functional validation of DIC GWAS genes (Fonoudi et al. 2024). Of these, 152 are expressed in our data. The number of overlapping genes in each response cluster was determined.

##### Heart failure risk gene analysis

Trait associations were downloaded from the NHGRI-EBI GWAS Catalog (Cerezo et al. 2025) on 11/13/2025 for heart failure (EFO_0003144), resulting in a list of 299 unique HF GWAS genes. We intersected these genes with our full list of 14,319 expressed genes, determining that 236 genes are expressed in our data. The number of overlapping HF GWAS genes in each response cluster was determined.

All custom scripts are available at https://github.com/mward-lab/Pfortmiller_DOX_recovery_2026 made possible by the workflowr package (Blischak et al. 2019).

## Results

### DOX induces transient DNA damage in cardiomyocytes

**Fig. 1.**
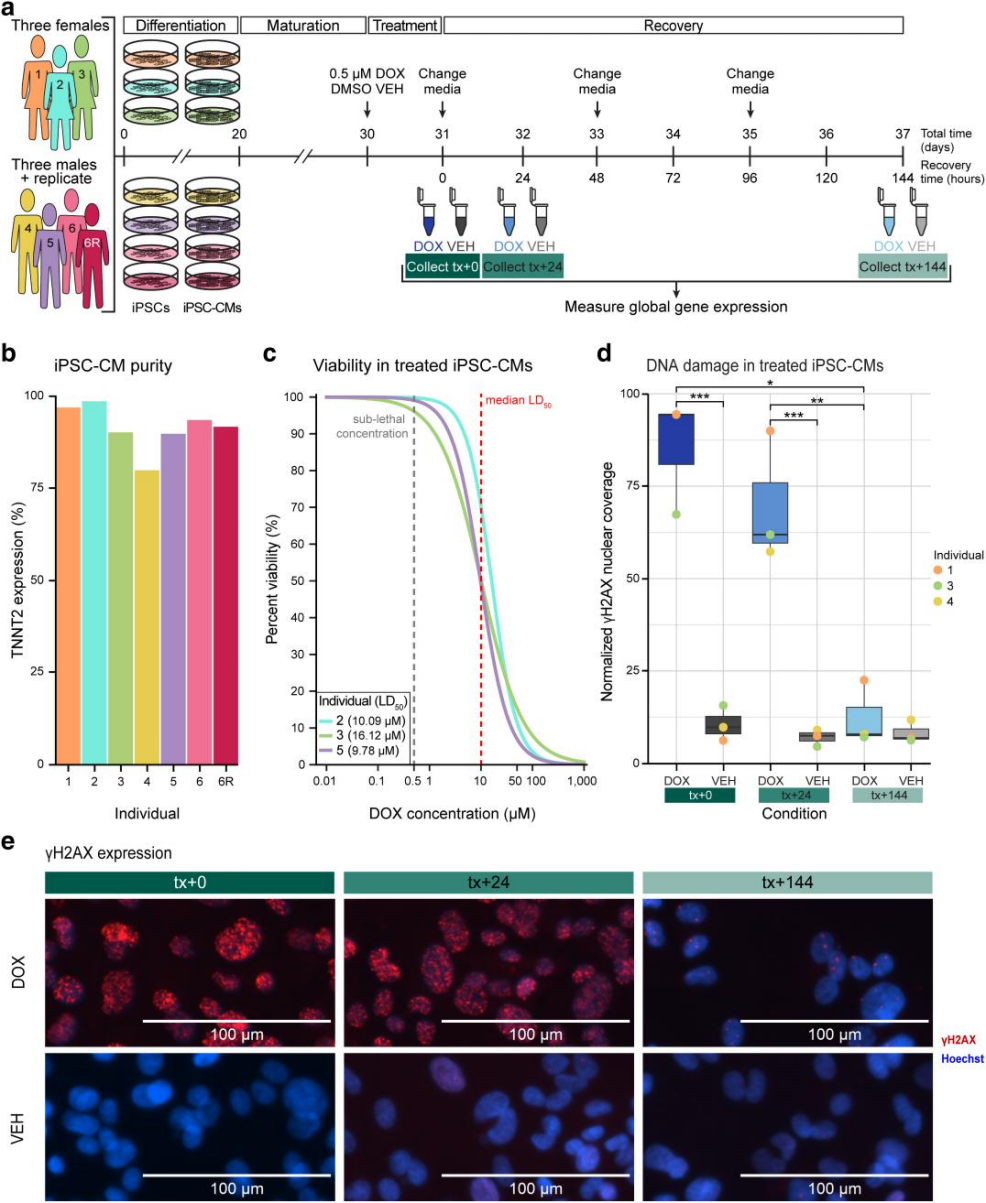
DOX induces DNA damage in iPSC-derived cardiomyocytes. a) Experimental design of this study. iPSCs derived from three females and three males aged between 18 and 32 were differentiated into cardiomyocytes (iPSC-CMs). iPSC-CMs were treated for 24 h with 0.5 μM doxorubicin (DOX) and a vehicle control (VEH) and then assayed directly following treatment (tx + 0), after 24 h of recovery (tx + 24), and after 144 h of recovery (tx + 144). b) Purity of differentiated iPSC-CMs as measured by flow cytometry for cardiac troponin T (TNNT2) expression. c) Percent viability of iPSC-CMs over increasing concentrations of DOX after 24 h of treatment. Cell viability after DOX treatment was assessed in three individuals (Individuals 2, 3, 5) and normalized to matched VEH. Dose–response curves were generated using a four-point log-logistic regression with the upper asymptote set to 1. LD_50_ values were calculated for each individual, with the median LD_50_ shown in red. The selected sublethal concentration for further experimentation is represented by the gray line (0.5 μM). d) Quantification of DNA damage as measured by γH2AX expression intensity in nuclei from DOX- and VEH-treated iPSC-CMs from three individuals (Individuals 1, 3, 4). Asterisks represent a statistically significant difference in γH2AX expression between DOX- and VEH-treated cells at each timepoint, as well as a statistically significant difference between DOX-treated cells across timepoints (*P < 0.05, **P < 0.01, ***P < 0.001, t-test). e) Immunofluorescence staining for the γH2AX marker (red) in iPSC-CM nuclei stained with Hoechst (blue) at tx + 0, tx + 24, and tx + 144 in DOX- and VEH-treated cells in a representative individual (Individual 1). Scale bar of 100 μm.

We differentiated iPSCs from three male and three female healthy donors into iPSC-CMs (Fig. 1a). We metabolically selected iPSC-CMs using lactate-containing media and matured the cells in glucose-free CMM media from Day 20 to Day 30 to promote oxidative phosphorylation, the primary metabolic pathway in adult cardiomyocytes (Rana et al. 2012). Flow cytometry analysis of cardiac troponin T (TNNT2) expression indicated a high proportion of cardiomyocytes across individuals (median purity of 91.8%; Fig. 1b). In order to investigate the direct effects of DOX treatment on iPSC-CMs over time, we first identified a sublethal drug concentration. We previously showed that the median LD50 of DOX-treated iPSC-CMs from six individuals following 48 h of treatment is 14.02 μM (Matthews et al. 2024). To determine the LD50 following 24 h of treatment, we treated iPSC-CMs from three representative individuals with a range of DOX concentrations (0.01 μM to 1,000 μM) and a DMSO vehicle (VEH) control for 24 h and determined the proportion of viable cells. We observed a dose-dependent decrease in viability following DOX treatment as expected (median LD50 = 10.09 μM; Fig. 1c). We selected a sublethal concentration of 0.5 μM DOX (greater than 90% viability following treatment across all three individuals) for all subsequent experiments.

To measure the effect of DOX on cardiomyocytes over time, we treated iPSC-CMs with 0.5 μM DOX and VEH for 24 h and assayed responses at three timepoints following treatment: (i) immediately prior to the removal of DOX and VEH treatment (tx + 0), (ii) 24 h posttreatment removal (tx + 24), and (iii) 144 h posttreatment removal (tx + 144). Given the known role of DOX in inducing DNA damage, we first asked whether DNA damage is induced in this system and second whether it is sustained following the removal of DOX. We quantified the effects of DOX on DNA damage by assaying the expression of the DNA double-stranded break marker γH2AX by immunofluorescence staining in three individuals. As expected, at tx + 0 there is an increase in DNA damage in DOX-treated iPSC-CMs compared to VEH (t-test; *P* < 0.001; Fig. 1d and e). The DNA damage is maintained at tx + 24 (*P* < 0.001). However, by tx + 144 there is no difference in the level of DNA damage between the DOX- and VEH-treated cells. These results are recapitulated when considering total γH2AX protein expression (Supplementary Fig. 1 in File 1). The pattern of total p53 expression matches that of γH2AX indicating that a robust DNA damage response (DDR) is induced (Supplementary Fig. 1 in File 1). Neither nuclei content nor nuclei number change in response to DOX treatment or time suggesting that DNA content is not affected (Supplementary Fig. 2 in File 1). These results suggest that our model captures DOX-induced DNA damage that clears over time.

### Transcriptional response to DOX decreases over time

**Fig. 2.**
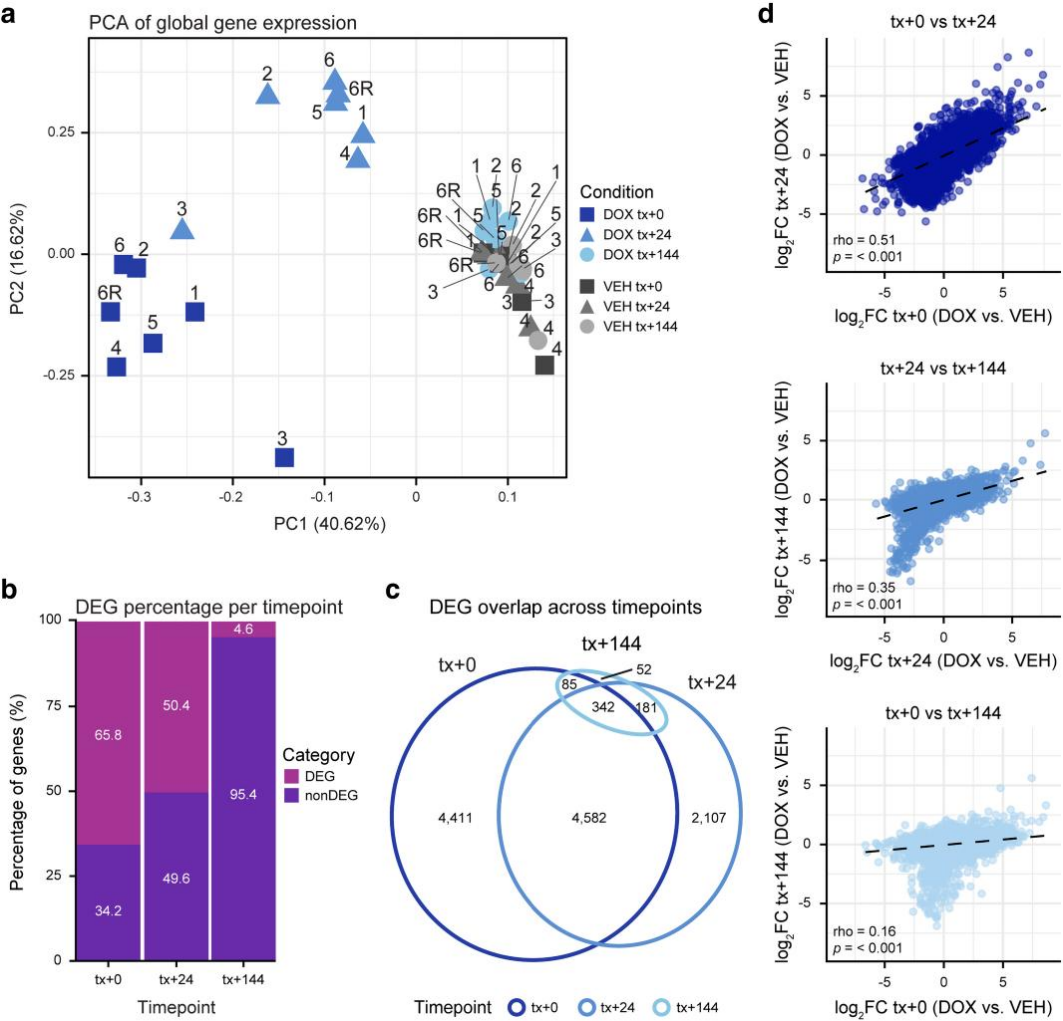
DOX induces global gene expression changes over time. a) Principal component analysis of RUV-corrected gene expression measurements across 42 samples from six individuals (1, 2, 3, 4, 5, 6) with a replicate of Individual 6 (6R) at treatment timepoints (DOX_tx + 0: dark blue; DOX_tx + 24: medium blue; DOX_tx + 144: light blue; DMSO_tx + 0: dark gray; DMSO_tx + 24: medium gray; DMSO_tx + 144: light gray). Data is representative of log_2_ cpm values of 14,319 expressed genes. b) Proportion of differentially expressed genes (DEGs) between DOX- and VEH-treated samples out of 14,319 expressed genes across timepoints. c) Overlap of DEGs at each timepoint. d) Spearman correlation (ρ) of log_2_ fold change response magnitude between timepoints.

We have previously shown that 0.5 μM DOX induces thousands of gene expression changes following 24 h of treatment in iPSC-CMs (Matthews et al. 2024). We therefore asked whether these changes are sustained over time. To do so, we treated iPSC-CMs from six individuals with 0.5 μM DOX and VEH and measured global gene expression by RNA-seq at tx + 0, tx + 24, and tx + 144. We replicated the differentiation and treatment for Individual 6 to account for any unknown technical variation. We processed the 42 samples in treatment-balanced batches across individuals (Supplementary Table 1 in File 2). All samples contained at least 20 million reads (Supplementary Fig. 3a in File 1), with mapping rates > 99.0% (Supplementary Table 2 in File 2). The read depth is similar across treatments (Supplementary Fig. 3b in File 1) and timepoints (Supplementary Fig. 3c in File 1). We removed lowly expressed genes resulting in a total of 14,319 expressed genes for further analysis. We accounted for unknown technical variation in the data using RUVs and the replicated Individual 6 (Risso et al. 2014). Following removal of one factor of unknown variation, PCA reveals that DOX treatment time is associated with the primary source of variation in the data (40.62%; Fig. 2a and Supplementary Fig. 4 in File 1). Similarly, samples cluster by treatment group, and those within the DOX treatment group are clustered by treatment time, unlike the VEH group (Supplementary Fig. 5 in File 1).

To identify DOX response genes at each timepoint, we performed pairwise differential expression analysis by comparing DOX and VEH treatments. We randomly selected one Individual 6 replicate and included the RUV-identified weight as a covariate in the linear model. We find thousands of differentially expressed genes (DEGs) at tx + 0 and tx + 24 and hundreds at tx + 144 (DOX vs VEH tx + 0 = 9,420; tx + 24 = 7,212; tx + 144 = 660; 5% FDR; Supplementary Fig. 6 in File 1; Supplementary Tables 3 to 5 in File 2). These changes correspond to 66% of all expressed genes being DEGs in tx + 0, 50% in tx + 24, and 5% in tx + 144 (Fig. 2b). While there is a roughly even distribution of DEGs that are up- and downregulated at tx + 0 and tx + 24 (48% upregulated for both), the majority of tx + 144 DEGs are downregulated (71%; *n* = 466; Supplementary Fig. 6 in File 1).

We then asked how many of the DEGs are shared across timepoints. We find that nearly half of all tx + 0 DEGs (49%; *n* = 4,582) are DEGs in tx + 24 and only 342 (4%) are DEGs in tx + 0, tx + 24, and tx + 144 (Fig. 2c). To compare global responses across conditions, we considered the magnitude of the treatment effect (log_2_ fold change) across all genes rather than just those that meet an FDR-based cutoff. There is a strong correlation between responses at early timepoints (tx + 0 vs tx + 24 ρ = 0.51; *P* < 0.001), which decreases over time (tx + 24 vs tx + 144 ρ = 0.35; *P* < 0.001; tx + 0 vs tx + 144 ρ = 0.16; *P* < 0.001 Fig. 2d). These findings suggest that most genes return to baseline expression levels 7 days after DOX treatment.

### Hundreds of response genes are sustained days after DOX treatment

**Fig. 3.**
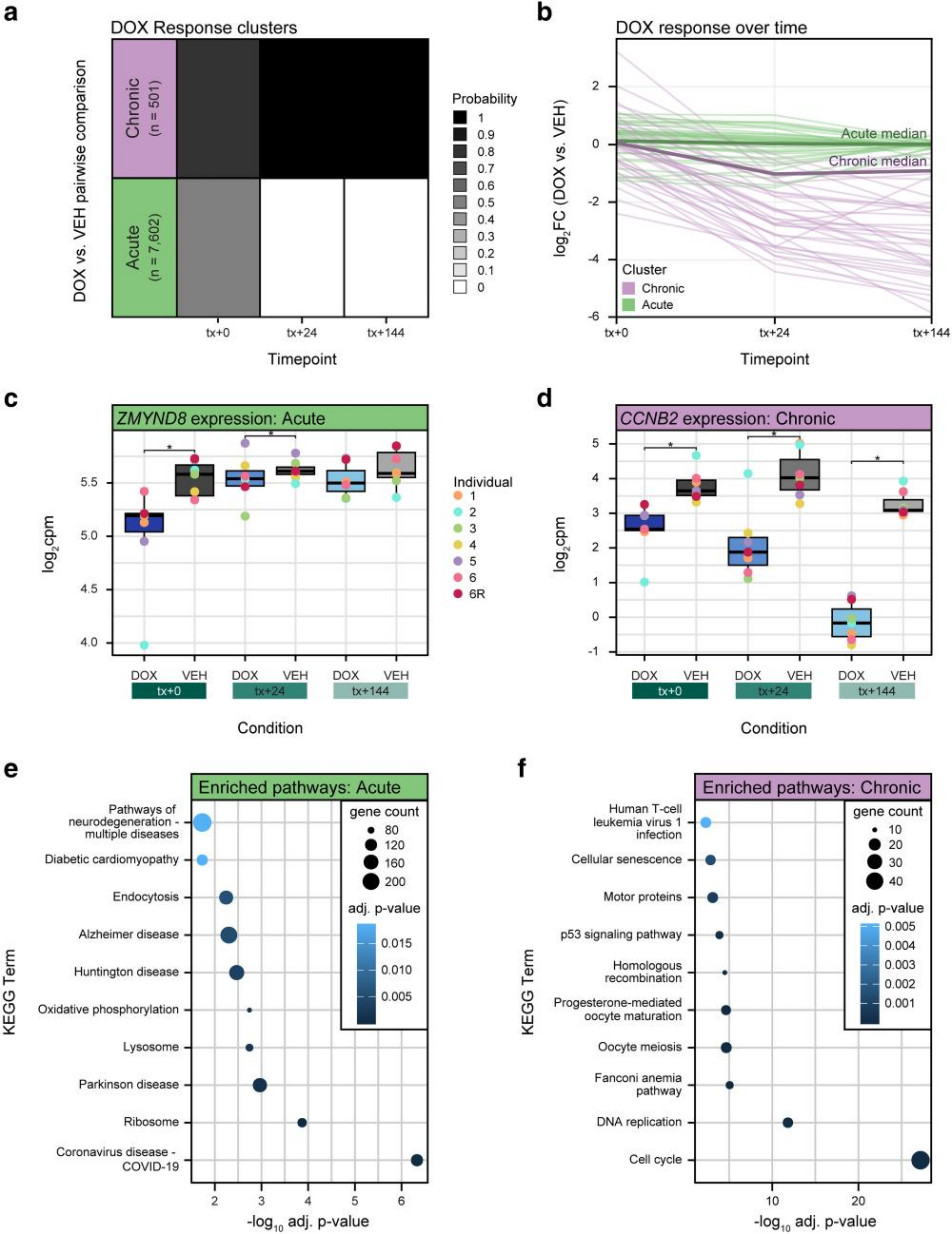
Trajectory analysis reveals acute and chronic DOX response genes. a) Gene expression clusters identified through joint modeling of test pairs over time. Shades of gray represent the posterior probabilities of genes being differentially expressed at each timepoint. Genes are categorized into two clusters based on their likelihood of belonging to a cluster and posterior probabilities: genes with posterior probability > 0.05 in tx + 0 and < 0.5 in tx + 24 and tx + 144 are designated as “acute response” (green), while those with posterior probability > 0.3 in tx + 0 and > 0.5 in tx + 24 and tx + 144 are designated as “chronic response” (purple). b) Log_2_ fold change of the top 100 genes from acute (green) and chronic (purple) response clusters. Median log_2_ fold change (bold line) for each cluster is shown. c) Gene expression (log_2_ cpm) of the *ZMYND8* acute response gene over time. Asterisks represent timepoints where the gene is classified as a DEG. d) Gene expression (log_2_ cpm) of the chronic response gene *CCNB2* over time. e) Biological pathways enriched in acute response genes. The top 10 KEGG terms that are significantly enriched in the acute gene set compared to all expressed genes are shown. Size of points indicates gene counts per term, while color scale represents adj. *P* value. f) Top 10 KEGG terms significantly enriched among chronic response genes.

To determine gene expression response dynamics over time, we used a Bayesian approach to jointly model pairs of tests at each timepoint (See Methods). Following BIC and AIC analysis, we find that the major expression patterns are explained by two response clusters (Supplementary Fig. 7 in File 1). The first cluster corresponds to genes that respond only in tx + 0 and not in tx + 24 or tx + 144, which we define as “acute response” genes (Fig. 3a; Supplementary Table 6 in File 2). The second cluster corresponds to genes that respond in all three timepoints, which we define as “chronic response” genes (Supplementary Table 7 in File 2). The majority of genes assigned to a cluster are classified as acute response genes (*n* = 7,602; 94% of 8,103 assigned genes). However, there are 501 chronic response genes. The absolute log_2_ fold change of genes in the acute response cluster approaches zero over time, while genes in the chronic response cluster do not (Fig. 3b). In fact, many of the chronic response genes increase in effect over time (Supplementary Fig. 8 in File 1). Individual genes assigned to these clusters show the expected expression patterns. For example, *ZMYND8* is a gene in the acute response cluster that is downregulated at tx + 0 (log_2_ fold change = −0.50) and tx + 24 (log_2_ fold change = −0.32) but not at tx + 144 (Fig. 3c). *CCNB2* in the chronic response cluster is increasingly downregulated across timepoints (Fig. 3d). Gene expression response magnitudes are independent of gene expression levels across the acute and chronic clusters suggesting that acute changes are not due to fluctuations in lowly abundant transcripts (Supplementary Fig. 9 in File 1). Fifty-five percent of DOX-responsive proteins previously identified following 24 h of 0.5 μM DOX treatment (174 of 319 proteins) (Johnson et al. 2025) are classified as acute response genes (one is classified as chronic) suggesting that short-lived mRNA changes have downstream consequences.

To identify biological pathways associated with each response cluster, we performed pathway enrichment analysis. The acute response cluster is enriched for terms related to the ribosome and lysosome as well as oxidative phosphorylation and endocytosis compared to all expressed genes (Fisher's one-tailed test; adj. *P* < 0.05; Fig. 3e; Supplementary Table 8 in File 2). The chronic response cluster is enriched for cell cycle (*CDKN1A*, *CDC20*, *E2F2*), DNA replication (*POLD3*, *FEN1*, *MCM2*), p53 signaling (*CCNB2*, *FAS*, *CDK1*), homologous recombination (*RAD51*, *BRCA1*, *BLM*), and cellular senescence pathways (*CDK1*, *CHEK1*, *MAP2K6*; adj. *P* < 0.05; Fig. 3f; Supplementary Table 8 in File 2). This suggests that the two response clusters represent distinct sets of genes associated with distinct cellular processes.

### Chronic response genes respond to treatment with other TOP2i

**Fig. 4.**
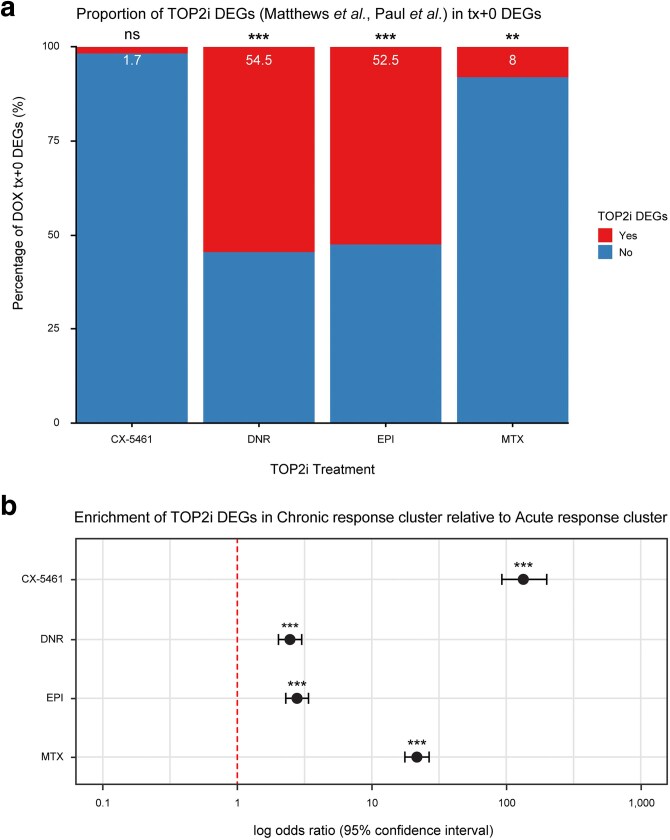
Chronic response genes are enriched in TOP2i DEGs compared to acute response genes. a) Proportion of tx + 0 DEGs that overlap DEGs following 24 h of treatment with other TOP2i (red). Data was obtained for CX-5461 from Paul et al. (Paul et al. 2026) and daunorubicin (DNR), epirubicin (EPI), and mitoxantrone (MTX) from Matthews et al. (Matthews et al. 2024). Significance of the overlap was determined by permutation testing (***P < 0.001). b) Enrichment of TOP2i DEGs in chronic response genes compared to acute response genes. A Fisher's exact test was used to calculate enrichment where odds ratios and 95% confidence intervals are plotted. Asterisk represents a significant enrichment (***P < 0.001).

As the primary mechanism of action of DOX is associated with inhibition of TOP2, we asked whether the response patterns we observe are recapitulated across other TOP2i drugs. We obtained DEGs from iPSC-CMs treated for 24 h with 0.5 μM TOP2i. 51.9% to 83.9% of tx + 0 DEGs are DEGs following DOX treatment in other studies (Matthews et al. 2024; Paul et al. 2026). The higher overlap set was from a study which included the same panel of individuals, while the lower overlap set had only three individuals in common and included female individuals only. As expected, anthracyclines showed the highest overlap with tx + 0 DEGs (54.5% for DNR and 52.5% for EPI), followed by the anthracenedione MTX (8.0%; Fig. 4a). The unrelated G-quadruplex inhibitor CX-5461 exhibited the lowest overlap (1.7%).

We then asked whether the chronic response genes are likely to be relevant across TOP2i. Indeed, we find that the majority of chronic response genes respond to other TOP2i (68.7% respond to DNR, 65.9% to EPI, 56.9% to MTX, and 40.5% to CX-5461; Supplementary Fig. 10 in File 1). Notably there is an enrichment of all TOP2i DEGs in the chronic response cluster compared to the acute response cluster (Fisher's exact test; *P* < 0.05; Fig. 4b). These results suggest that chronic response genes that do not recover from DOX treatment may be relevant to TOP2i more broadly.

### Chronic response genes are enriched for DNA damage and senescence-associated genes

**Fig. 5.**
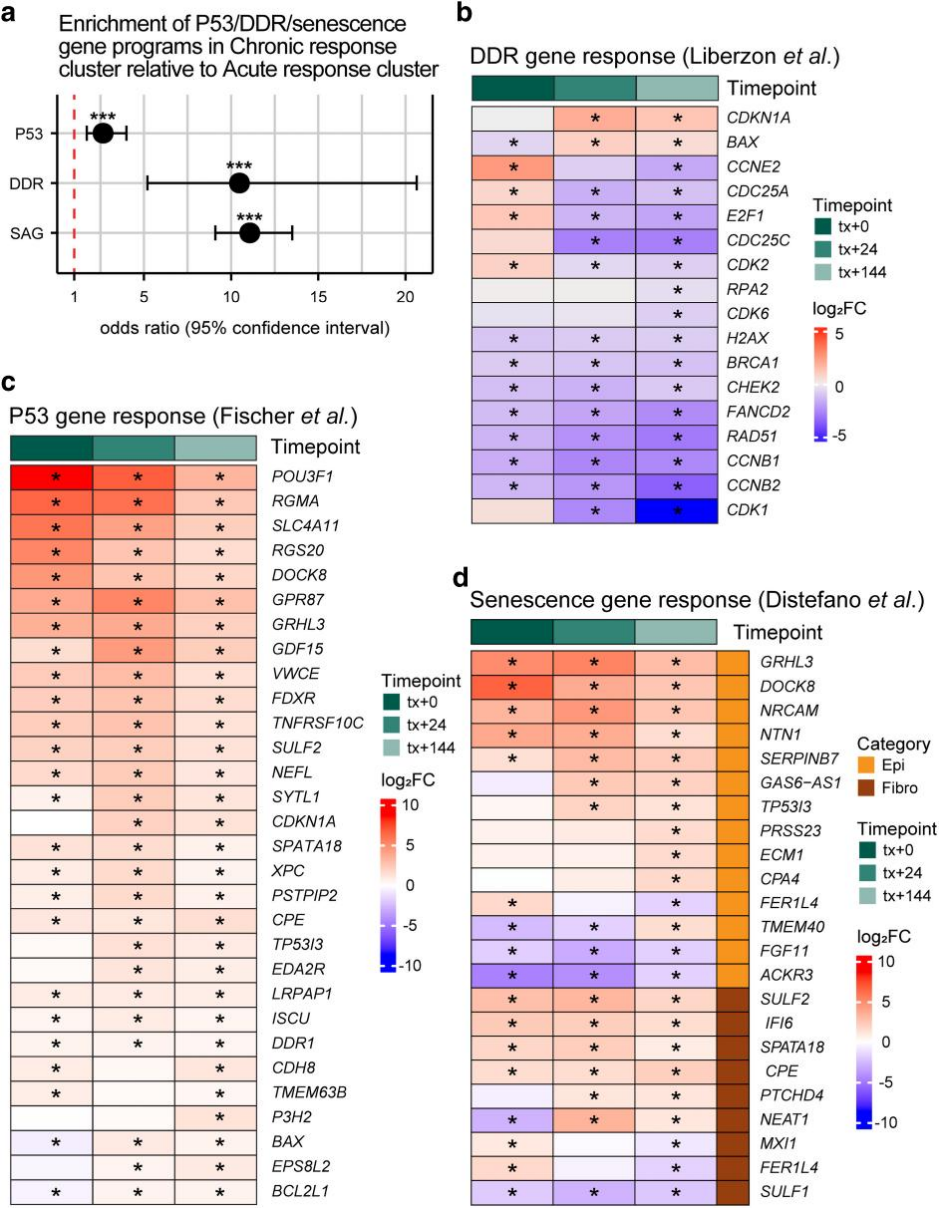
Chronic response genes are enriched for DNA damage-associated genes. a) Enrichment of 66 DDR genes (Liberzon et al. 2015), 346 p53 target genes (Fischer 2017), and 2,497 senescence-associated genes (DePianto et al. 2021; Distefano et al. 2023) in chronic response genes compared to acute response genes. Asterisk represents a significant enrichment as calculated by Fisher's exact test (***P < 0.001). Odds ratios and 95% confidence intervals are plotted. b) Gene expression response (log_2_ fold change) across timepoints of DDR genes that are classified as DEGs at tx + 144. Color scale represents either upregulation (red), no change (white), or downregulation (blue). Asterisks represent genes that are classified as DEGs in each respective timepoint. c) Gene expression response (log_2_ fold change) across timepoints of p53 response genes that are DEGs at tx + 144. d) Gene expression response (log_2_ fold change) across timepoints of senescence-associated genes classified as DEGs at tx + 144 identified in epithelial cells (orange) and fibroblasts (brown) (DePianto et al. 2021).

Given that we observed DOX-induced DNA damage that was restored over time at the cellular level, we asked whether genes known to be relevant to the response to DNA damage are among the chronic response gene set. First, we obtained a set of 66 genes associated with the DDR (Liberzon et al. 2015) and calculated the proportion of DDR genes in the acute and chronic response clusters. We find that the chronic response cluster is enriched for DDR genes compared to the acute response cluster (Fisher's exact test; OR = 10.4, confidence interval (CI) = 5.1 to 20.5; *P* < 0.001; Fig. 5a). Second, we obtained a set of 346 genes identified as transcriptional targets of the DNA damage-associated transcription factor p53 (Fischer 2017). Again, we find that the chronic response cluster is enriched for p53 target genes compared to the acute response cluster (Fisher's exact test; OR = 2.5, CI = 1.6 to 3.9; *P* < 0.001; Fig. 5a).

In line with DNA damage-associated genes being enriched in the chronic response cluster, 11 of 65 DDR genes are classified as DEGs in all timepoints including *CCNB2*, *FANCD2*, and *RAD51* that relate to cell cycle and repair pathways (Fig. 5b). DNA damage response genes are enriched in tx + 144 DEGs compared to non-DEGs at this timepoint (Fisher's exact test; OR = 7.5; 95% CI = 4.0 to 13.3; *P* < 0.05; Supplementary Fig. 11a in File 1); however, DDR genes are not enriched in the earlier timepoints. We also find that many p53 target genes are DEGs in each timepoint (82 of 300) including *NEFL*, *XPC*, and *BAX* (Fig. 5c). p53 target genes are enriched in DEGs at all timepoints (Fisher's exact test; tx + 0: OR = 1.5; 95% CI = 1.1 to 1.9; tx + 24: OR = 2.1, CI = 1.6 to 2.8; tx + 144: OR = 2.3; CI = 1.5 to 3.4; *P* < 0.05; Supplementary Fig. 11b in File 1). Together, these results suggest that the genes that do not recover from DOX treatment relate to DNA damage in line with them being enriched in p53 and cell cycle associated pathways.

Low doses of DOX treatment in cardiomyocytes have been shown to induce a senescent-type state with characteristic beta galactosidase activity (Maejima et al. 2008), similar to its effects on proliferating cells. We therefore investigated whether a collated set of senescence-associated genes from fibroblasts and epithelial cells (DePianto et al. 2021; Distefano et al. 2023) contribute to the chronic response. We find that the chronic response cluster is enriched for senescence-associated genes compared to the acute response cluster (Fisher's exact test; OR = 11, CI = 9.0 to 13.4; *P* < 0.001; Fig. 5a). Senescence-associated genes that are upregulated DEGs at tx + 144 include *GRHL3*, *TP53I3*, and *CPA4* (Fig. 5d). Senescence-associated genes are enriched in tx + 24 and tx + 144 DEGs compared to non-DEGs at these timepoints (tx + 24 Fisher's exact test; OR = 1.6; 95% CI = 1.5 to 1.8; *P* < 0.001; tx + 144: OR = 6.5; 95% CI = 5.5 to 7.6; *P* < 0.001 Supplementary Fig. 11c in File 1); however, they are not enriched in the tx + 0 timepoint. These results suggest that DOX induces senescence in these cardiomyocytes over time.

### Over 100 genes show increased response to DOX over time

**Fig. 6.**
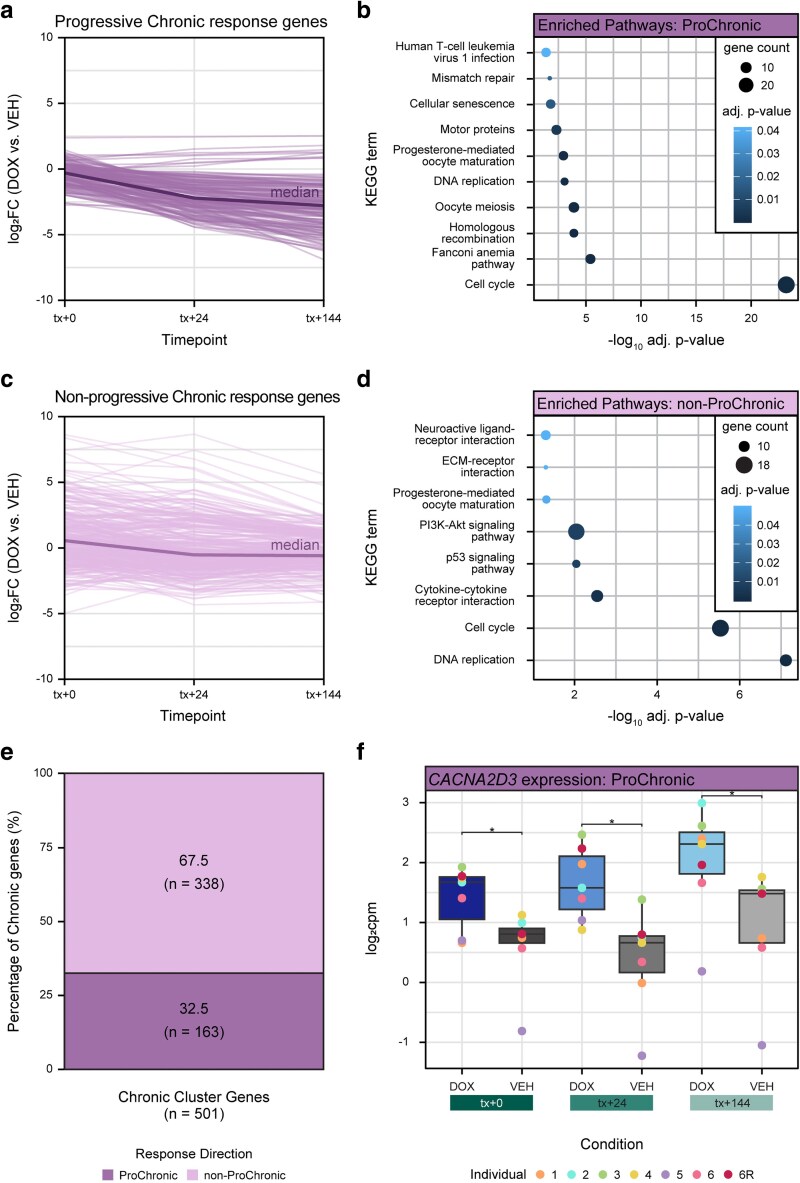
Over a hundred genes continue to diverge from baseline 7 days posttreatment. a) Log_2_ fold change of chronic response genes that continue to diverge from baseline expression over time (progressively chronic). Median log_2_ fold change (bold line) is shown. b) Biological pathways enriched in progressively chronic response genes. The top 10 KEGG terms that are significantly enriched in the progressively chronic gene set compared to all expressed genes are shown. Color scale represents adj. *P* value, while point size indicates the number of genes in each term. c) Log_2_ fold change of chronic response genes that do not continue to diverge from baseline expression over time. Median log_2_ fold change (bold line) is shown. d) Biological pathways enriched in chronic response genes that do not progressively increase in response. The top 10 KEGG terms that are significantly enriched in this gene set compared to all expressed genes are shown. e) Percentage of chronic response genes that are progressively chronic and not. f) Gene expression (log_2_ cpm) of the *CACNA2D3* progressively chronic response gene over time. Asterisks represent timepoints where the gene is classified as a DEG.

As we noted above, many chronic response genes show increased response to DOX over time. We therefore further stratified the 501 genes within the chronic response cluster to isolate the set where the response to DOX increases over time (absolute log_2_ fold change tx + 0 < absolute log_2_ fold change tx + 24 < absolute log_2_ fold change tx + 144; Supplementary Table 7 in File 2). This yielded 163 progressively chronic response genes (Fig. 6a). Ninety-four percent of these genes are progressively downregulated. These genes are enriched for cell cycle (eg *CDC20*, *ORC1*, *KNL1*) and DNA repair processes (*RAD51*, *EXO1*, *MCM7*) (Fig. 6b & Supplementary Table 8). This is in contrast to genes that are chronic, but do not increase in response over time, that are uniquely enriched for processes related to ligand–receptor interactions (*TNFRSF9*, *ITGA8*, *EDNRB*), p53 (*FAS*, *CHEK1*, *CDKN1A*), and PI3K–Akt signaling (*NGF*, *CDK2*, *FGFR3*) (Fig. 6c and d & Supplementary Table 8 in File 2). Overall, 33% of chronic response genes are progressively responsive (Fig. 6e).

There are nine genes that are progressively upregulated. These include *ASTN1*, *EPS8L2*, *CEMIP*, *P3H2*, *CACNA2D3*, *PDE11A*, *ACHE*, *CPE*, and *PTCHD4* (Table 1*)*. *CACNA2D3* is the target of amlodipine, an approved drug for hypertension and angina (Fig. 6f). Blocking *CACNA2D3* with amlodipine has been shown to inhibit DOX-induced apoptosis in neonatal rat cardiomyocytes (Yamanaka et al. 2003). These results suggest that there are a small number of genes that continue to diverge in expression from baseline 7 days following treatment.

**Table 1.** Chronic response genes include candidate toxicity biomarkers and disease genes.

Gene	Response cluster	Direction (DOX vs VEH log_2_FC)	tx + 0 DEG	tx + 24 DEG	tx + 144 DEG	Biomarker candidate	Heart failure gene
*ASTN1*	ProChronic	up_up_up	No	No	No	Yes	No
*EPS8L2*	ProChronic	down_up_up	No	Yes	Yes	Yes	No
*CEMIP*	ProChronic	up_up_up	No	Yes	No	Yes	No
*P3H2*	ProChronic	up_up_up	No	No	Yes	Yes	No
*CACNA2D3*	ProChronic	up_up_up	Yes	Yes	Yes	Yes	No
*PDE11A*	ProChronic	up_up_up	No	Yes	Yes	Yes	No
*ACHE*	ProChronic	up_up_up	Yes	Yes	Yes	Yes	No
*CPE*	ProChronic	up_up_up	Yes	Yes	Yes	Yes	No
*PTCHD4*	ProChronic	down_up_up	No	Yes	Yes	Yes	No
*ABCA12*	non-ProChronic	up_up_up	Yes	Yes	No	No	Yes
*CDKN1A*	non-ProChronic	up_up_up	No	Yes	Yes	No	Yes
*CIT*	non-ProChronic	down_down_down	Yes	Yes	Yes	No	Yes
*TRAIP*	non-ProChronic	down_down_down	Yes	Yes	Yes	No	Yes

### Most DIC genes are acute response genes

**Fig. 7.**
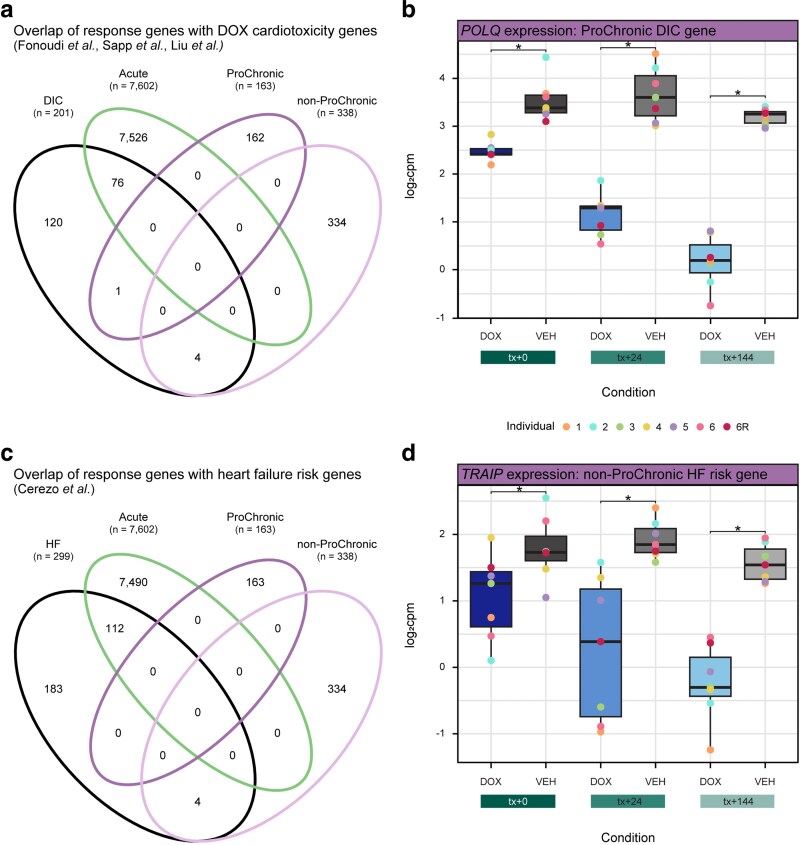
Chronic DOX response genes are relevant to DIC and heart disease. a) Overlap between DIC cardiotoxicity genes (functionally validated DOX targets [Fonoudi et al. 2024] and CRISPRi targets [Sapp et al. 2021; Liu et al. 2024]) and acute, progressively chronic and nonprogressively chronic response genes. b) Gene expression (log_2_ cpm) of the DIC gene *POLQ* that is classified as a progressively chronic response gene over time. Asterisks represent timepoints where the gene is classified as a DEG. c) Overlap between heart failure risk loci genes (Cerezo et al. 2025) and acute, progressively chronic and nonprogressively chronic response genes. d) Gene expression (log_2_ cpm) of the heart failure gene *TRAIP* that is classified as a chronic response gene over time.

DOX treatment is associated with DIC in some patients. GWAS has identified tens of genetic variants associated with risk for developing cardiotoxicity following DOX treatment (Schneider et al. 2017; Park et al. 2020). Twenty-five genes in these loci have been functionally validated in in vitro models (Fonoudi et al. 2024). Large-scale CRISPR/Cas9 screens in iPSC-CMs have also identified genes which mediate the effects of DOX (Sapp et al. 2021; Liu et al. 2024). We therefore combined the sets of genes identified by these studies into a DIC gene set consisting of 201 genes expressed in our iPSC-CMs and investigated their longitudinal response to DOX. Of the 81 genes that are classified in response signatures, 94% are classified as acute response genes (*n* = 76; Fig. 7a). One gene is classified as progressively chronic, *POLQ*. This gene, which encodes polymerase theta, is important in rapid but low fidelity DNA repair (Wood and Doublie 2016). Its expression is progressively downregulated over time (Fig. 7b). Four genes are classified in the broader chronic response set: *CPMK2*, *HUNK*, *TAGLN2*, and *COL19A1*.

Given that DIC can lead to heart failure, we obtained the set of genes in heart failure risk loci (Cerezo et al. 2025). Of the 116 genes that are classified as response genes, 97% are classified as acute response genes (*n* = 112; Fig. 7c). There are no progressively chronic genes associated with heart failure but four including *ABCA12*, *CDKN1A*, *CIT*, and *TRAIP* are in the chronic set that is not progressively different over time (Table 1). *TRAIP* is downregulated in response to DOX and is known to be involved in the DDR (Fig. 7d) (Harley et al. 2016).

## Discussion

DOX-induced cardiotoxicity is a well-established phenomenon in cancer patients treated with DOX. Many studies have indicated widespread effects on global gene expression in human cardiomyocytes following acute exposure to DOX and linked these effects to the phenotype in humans (Burridge et al. 2016; Knowles et al. 2018; Matthews et al. 2024). Here, we measured the effects of DOX treatment and recovery from treatment to mimic chronic effects of the drug. We identified thousands of gene expression changes that diminish over time. However, we also identified a chronic response signature corresponding to p53 activation and suppression of DNA repair.

By measuring gene expression trajectories following DOX treatment and removal, we identified two predominant response signatures—genes that respond to treatment and return to baseline levels of expression following 24 h of recovery and genes that respond to treatment and sustain their response following six days of recovery. The vast majority of response genes return to baseline during recovery (94%); however, there are 501 genes that show perturbed expression throughout the recovery period. Our results are in line with prior studies that have compared the effects of DOX treatment and washout in human-derived cardiomyocytes and determined that most response genes return to baseline levels of expression a week following treatment removal (Holmgren et al. 2015; Reyes et al. 2018; McSweeney et al. 2019). By considering iPSC-CMs from multiple individuals and jointly modeling expression changes over time, we were able to robustly identify both acute and chronic response genes.

Chronic response genes are enriched for DDR pathways and p53 signaling. p53 target genes continue to be activated over time, while DDR genes are progressively downregulated. However, we observe that DNA damage, as measured by the expression of γH2AX, is diminished at the final timepoint suggesting that double-strand breaks have been resolved. The discrepancy between observed DNA damage and DDR gene mRNA changes may suggest a chronic stress state or secondary effects of the DOX treatment. For example, there may be oxidative DNA damage initiated through drug-induced reactive oxygen species (Yoshida et al. 2009); however, classic markers of oxidative DNA damage including *OGG1*, *PARP1*, and *ATM* are DEGs in early timepoints only.

We observe a significant enrichment in senescence-associated genes among chronic response genes. Low doses of DOX induce senescence in proliferating cells by inhibiting DNA replication. Cardiomyocytes are postmitotic and undergo limited replication. More than 90% of iPSC-CMs are mononucleoid and more than 80% are diploid, suggesting that endoreplication is not a major contributor to iPSC-CM function (Woo et al. 2019). Similarly, less than 10% of this cell type have been shown to be able to incorporate EdU further suggesting limited replication ability (Woo et al. 2019). Our results suggest that senescent programs converge in these different cell types where DOX has differing mechanisms of action. However, when considering a core senescence signature consisting of 55 senescence-associated genes identified from a range of cell types, excluding cardiomyocytes, we find only one gene, *PLK3*, to be within our set of chronic response genes (Hernandez-Segura et al. 2017). Senescence-associated phenotype markers including *IL6*, *IL8*, *MMP3*, and *CXCL1* are not expressed in our cardiomyocytes; however, *GDF15* is and is upregulated at all timepoints. Senescence has been observed in the hearts of patients treated with DOX, and *GDF15* is increased following a 28-day DOX treatment schedule in engineered heart tissue (Linders et al. 2023).

We note that the chronic response genes are largely distinct from genes that have previously been associated with DIC through global CRISPRi screening or CRISPRi targeted to genes in DIC-associated loci (Sapp et al. 2021; Fonoudi et al. 2024; Liu et al. 2024). This suggests the identification of a set of genes for further investigation as potential biomarkers or therapeutic targets. Indeed, we identify *CACNA2D3* as a gene that is progressively upregulated over time. This gene is important to cardiac function, and drugs that target it are used in the treatment of angina.

The limitation of iPSC-derived cardiomyocytes is that they resemble fetal cardiomyocytes more than adult cardiomyocytes. It is possible that the response to DOX and the DNA repair mechanisms differ between adult cardiomyocytes and iPSC-derived cardiomyocytes. To mitigate this potential effect, we used 30-day iPSC-CMs cultured in media to shift the metabolic state of the iPSC-CMs to be reflective of adult cardiomyocytes. We note however that in humans, cardiomyocytes proliferate up until age 20 (Mollova et al. 2013). Given that there are many pediatric cancers that are treated with DOX, we believe that this system is physiologically and clinically relevant.

In summary, we measured the effects of treatment and recovery from DOX in cardiomyocytes from six individuals. We found that DOX induces DNA damage, which is resolved following six days of recovery from treatment. Similarly, large-scale effects on gene expression are induced following treatment that diminish over time. However, there are a set of 501 genes that are persistently perturbed 6 days following treatment that are related to p53 signaling, DNA damage, and senescence. Our results suggest that chronic modeling of DOX treatment can highlight genes that may relate to the long-term effects of DOX treatment on the heart.

## Supplementary Material

jkag144_Supplementary_Data

## Data Availability

All RNA-seq data have been deposited in the Gene Expression Omnibus (www.ncbi.nlm.nih.gov/geo/) under accession number GSE313746. All custom analysis scripts are available at https://github.com/mward-lab/Pfortmiller_DOX_recovery_2026. Supplementary material includes File 1 incorporating Supplementary Figs. 1 to 11 and File 2 incorporating Supplementary Tables 1 to 8. Supplemental material available at G3 online.
